# Lack of association between leptin concentrations and cystic fibrosis: A meta-analysis and regression

**DOI:** 10.3389/fendo.2023.1126129

**Published:** 2023-03-13

**Authors:** Hong Qi, Hairong Liu, Pengcheng Zheng, Jie He

**Affiliations:** ^1^ Clinical Medical College of Chengdu Medical College, Chengdu, Sichuan, China; ^2^ Department of Geriatric Medicine, The First Affiliated Hospital of Chengdu Medical College, Chengdu, Sichuan, China; ^3^ Department of Pulmonary and Critical Care Medicine, The First Affiliated Hospital of Chengdu Medical College, Chengdu, Sichuan, China

**Keywords:** leptin, cystic fibrosis, systematic review, meta-analysis, bioinformatics analyses

## Abstract

**Background:**

Leptin (LEP) acts as a proinflammatory cytokine and may play an important role in the pathophysiology of cystic fibrosis (CF). This review aimed to assess the quantitative difference in leptin status between CF patients and non-CF controls.

**Methods:**

In this study, the researchers conducted systematic searches of various databases, such as PubMed, Excerpta Medica Database, Google Scholar, Web of Science, and the China National Knowledge Infrastructure. The data collected from the above databases were assessed using the Stata 11.0 and R 4.1.3 software. The correlation coefficients and the Standardized Mean Differences (SMD) were employed to assess the effect size. A combination analysis was also carried out with the help of either a fixed-effects or random-effects model. In addition, the single-cell sequencing GSE193782 dataset was obtained to determine the mRNA expression levels of LEP and leptin receptor (LEPR) in the bronchoalveolar lavage fluid, to verify the different leptin expression between the CF patients and healthy controls.

**Results:**

A total of 919 CF patients and 397 controls from 14 articles were included in this study. CF patients and non-CF controls showed similar serum/plasma leptin levels. Gender, specimen testing, age, and study design were all taken into account for carrying out subgroup analyses. The results revealed no variations in serum/plasma leptin levels between the controls and CF patients in the various subgroups. Female CF patients exhibited higher leptin concentrations compared to male CF patients, and male healthy individuals showed lower leptin levels than female healthy participants. Aside from the fact that serum/plasma leptin appeared to be favorably linked to fat mass and BMI, the findings in this study also indicated that serum/plasma concentrations were not associated with Forced Expiratory Volume in the first second (FEV1). No statistically significant differences were observed in the leptin and leptin receptor mRNA expression levels between the healthy controls and CF patients. The leptin receptor and leptin expression levels in alveolar lavage fluid were low in various cells, without any distinctive distribution patterns.

**Conclusions:**

The current meta-analysis indicated the absence of significant differences in leptin levels between CF patients and healthy individuals. Gender, fat mass, and BMI may all be correlated with leptin concentrations.

**Systematic review registration:**

https://www.crd.york.ac.uk/prospero/, identifier CRD42022380118.

## 1 Introduction

Cystic fibrosis (CF) is described as a life-shortening, multisystem genetic disease with an autosomal recessive inheritance pattern, which is attributed to mutations occurring in the cystic fibrosis transmembrane conductance regulator (*CFTR*) gene (1, 2). This disease affects >70,000 persons across the globe (3). CFTR is primarily responsible for controlling bicarbonate anions and chloride ions (4). The submucosal and airway mucosal glands typically express CFTR in their epithelial cells. A malfunctioning CFTR leads to infections and airway inflammation, a gradual decrease in lung function, eventually leading to respiratory failure and early death (5). Additionally, the CFTR gene is expressed in several physiological systems and other organs such as the lung, pancreas, skeletal muscle, gastrointestinal tract, cardiovascular, sweat glands, reproductive organs, etc (6). Consequently, CF is considered a complex multisystem disease, necessitating regular and comprehensive examinations to monitor and manage the occurrence of disease complications.

The adipocyte-derived protein hormone, i.e., leptin, helps to maintain homeostasis (7). Leptin, along with other adipokines, contributes to the inflammatory state occurring in obese individuals and causes both direct and indirect consequences that result in type 2 diabetes, metabolic syndromes, and cardiovascular diseases (8). Many studies have highlighted the important role played by leptin in CF. Leptin triggers inflammation in the lungs, and increased leptin levels have been linked to the progression and severity of symptoms in the presence of lung inflammatory stimuli (9, 10). Additionally, the correlation between leptin and fat accumulation may contribute to poor weight loss or gain in CF patients (11). In their study, Galiniak et al. (12) observed higher fasting leptin levels in the CF patients compared to healthy controls, and this difference may have resulted in patients’ impaired lung function. Arumugam et al. (13) reported no significant variations in the blood leptin levels between CF patients and healthy controls, in contrast to the study conducted by Polito et al. (9), who observed that leptin levels in healthy controls were significantly higher compared to those in CF patients. Since no meta-analysis has been conducted on leptin and CF, the correlation between CF and leptin concentrations could not be determined.

After considering the sporadic research conducted in the past, the meta-analysis and regression analysis were carried out to evaluate the link between CF and leptin concentrations, thus offering new insights into determining the etiology of CF.

## 2 Experimental procedures

### 2.1 Protocol registration

The protocol employed in this study was registered on PROSPERO **(**CRD42022380118; https://www.crd.york.ac.uk/prospero/display_record.php?RecordID=380118).

### 2.2 Eligibility criteria used in this review

The studies that determined the plasma and serum leptin concentrations in CF patients and compared them with the non-CF control patients, were included in this review. Case-control studies and intervention trials with a case-control comparison at baseline were both included in the population-based studies. A clinical diagnosis of CF must have been supported by an abnormal sweat test, diagnostic genotyping, and/or the participant being recruited through a CF clinic. The leptin concentrations of all the study participants, who were deemed clinically stable and free of signs of an acute respiratory exacerbation, were tested. The Shwachman score was used to categorize CF severity into three categories: mild disease (71-100 points), moderate disease (41-55 points), and severe disease (<41 points) (14). Reviews, case reports, and letters to the editor were not included for further analysis. The studies that compared data from CF patients with normative values published in the literature were disregarded. There were no restrictions on the publication period. The studies could be published in advance, included online texts, and were published in any language.

### 2.3 Source of information

Several databases, including Google Scholar, Web of Science, PubMed, Excerpta Medica database (EMBASE), and China National Knowledge Infrastructure (CNKI), were thoroughly investigated to identify studies conducted up to 12^th^ December 2022.

### 2.4 Data searches

The current study applied a 3-part search strategy to find studies that met eligibility requirements, similar to other systematic reviews conducted in this field (15). The primary strategy used in the review used a subject term or Medical Subject Heading (MeSH), in the following manner: “cystic fibrosis “[MeSH] AND (leptin [MeSH]). The extracted data was used for conducting Stage 2 of the literature review.

A different technique determined the references related to specific words in the abstract and/or title (ti/ab) sections. During the search process, the biological molecules and leptin that were determined in the MeSH search were AND-linked to “cystic fibrosis” in the following manner: “cystic fibrosis” [ti/ab] AND “leptin” [ti/ab] AND (blood [ti/ab] OR plasma [ti/ab] OR serum [ti/ab] OR “whole blood” [ti/ab]). To ensure a thorough, targeted, and sensitive search, the MeSH search was repeated at this point. In addition, the authors manually reviewed the conference proceedings, clinical trial registers, and the bibliographies of the studies and reviews that were included.

### 2.5 Study selection

**Figure 1:**
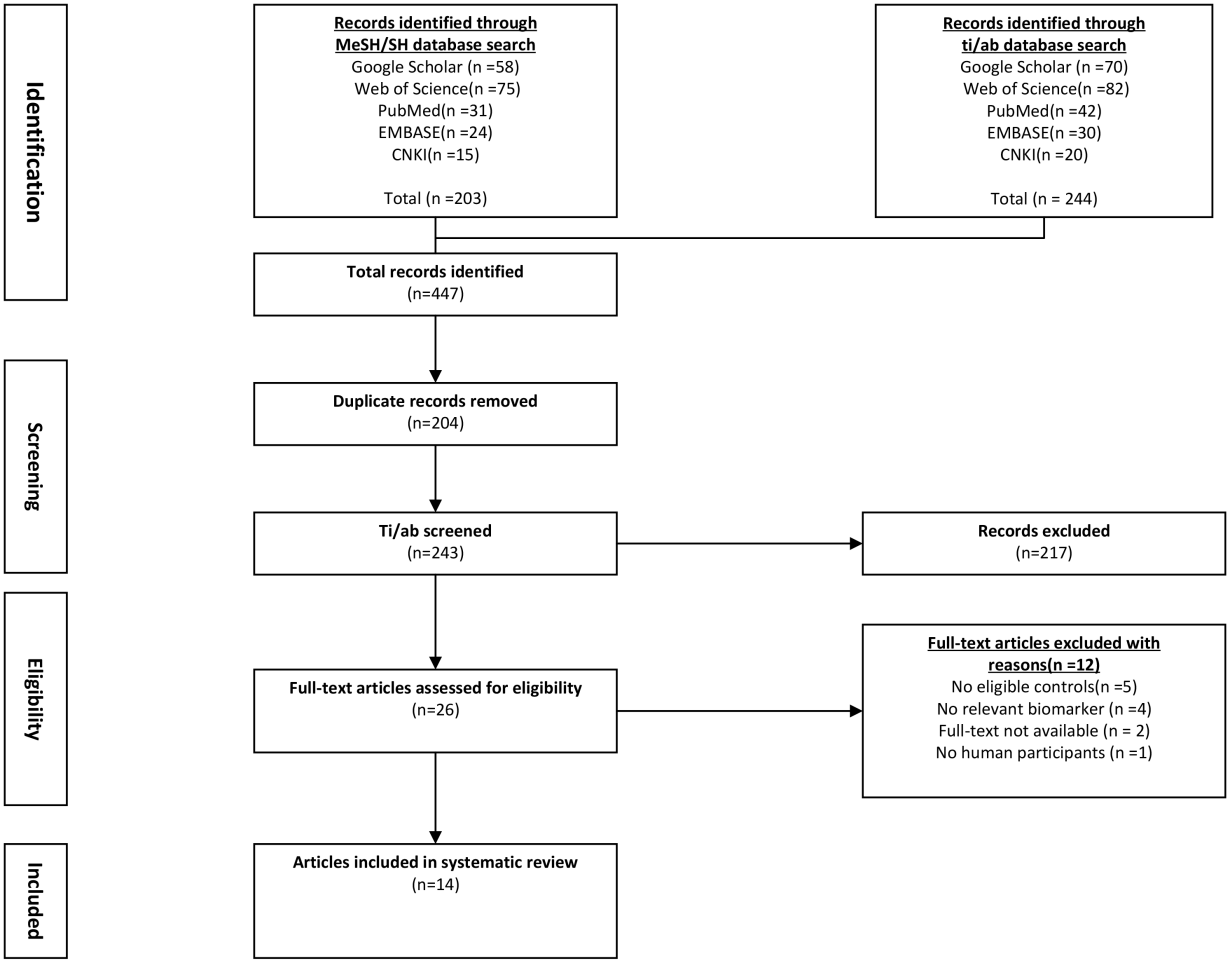
Flowchart of literature screening and selection process. CNKI, China National Knowledge Infrastructure; EMBASE, Excerpta Medica Database.

Two authors independently conducted the two steps of the screening and selection process. After eliminating the duplicate articles, it was determined whether the study fulfilled the eligibility requirements by screening the ti/ad sections of all references and excluding any that did not. Subsequently, the full texts of other references were downloaded and scrutinized using eligibility standards. The meta-analysis included all eligible references (**Table 1**). If the leptin concentrations were determined using two different references in the same study, the recent reference was used for meta-analysis. The PRISMA flow diagram (**Figure 1**) describes an overview of all findings derived from the article selection procedures.

**Table 1:** Characteristics of included studies.

Author	Year	Country	Sex(F/M)		Age(year)		BMI (kg/m2)		Leptin source	Assay approach	Study design
			Case	Control	Case	Control	Case	Control			
Galiniak S	2022	Poland	17/21	10/6	19.85±7.9	19.25±7.3	19.89±2.8	22.47±2.5	Serum	ELISA	Cross-sectional study
Granados A(NGT)	2021	USA	4/5	8/7	16±3	17±4	NA	NA	Plasma	RIA	Cross-sectional study
Granados A(AGT)	2021	USA	17/20	8/7	15±3	17±4	NA	NA	Plasma	RIA	Cross-sectional study
Granados A(CFRD)	2021	USA	8/5	8/7	17±3	17±4	NA	NA	Plasma	RIA	Cross-sectional study
Polito R	2019	Italy	43/55	57/59	31.1±7.6	30.9±8.7	23.4±3	22.4±3.6	Serum	ELISA	Case-control study
Nowak JK	2020	Poland	29/27		19.09±7.99		18.25±3.80		Serum	ELISA	
Monajemzadeh M	2013	Iran	20/23	17/26	3.43±3.16	3.87±3.46	15.1±0.5	21.6±0.6	Plasma	ELISA	Case-control study
Ziai S(NGT)	2012	Canada	20/29	10/7	27.14±8.05	25.28±3.97	21.36±2.72	22.39±1.69	Plasma	RIA	Case-control study
Ziai S(IGT)	2012	Canada	11/10	10/7	29.59±9.11	25.28±3.97	21.61±3.29	22.39±1.69	Plasma	RIA	Case-control study
Ziai S(CFRD)	2012	Canada	6/5	10/7	30.82±8.56	25.28±3.97	21.36±3.12	22.39±1.69	Plasma	RIA	Case-control study
Olveira G	2012	Spain	50/50	23/20	32.7±4	25.1±8.4	21.3±3.6	22.9±1.8	Serum	ELISA	Cross-sectional study
Speeckaert MM	2008	Belgium	53/63		14.95±11.26		NA	NA	Serum	ELISA	Case-control study
Cohen RI(mild)	2008	USA	9/10	10/10	25.36±7.21	29.35±2.39	23.37±2.6	22.64±2.39	Plasma	ELISA	Case-control study
Cohen RI(moderate)	2008	USA	16/14	10/10	31.64±8.56	29.35±2.39	22.57±2.88	22.64±2.39	Plasma	ELISA	Case-control study
Cohen RI(severe)	2008	USA	10/15	10/10	36.15±17.29	29.35±2.39	20.28±4.72	22.64±2.39	Plasma	ELISA	Case-control study
Boguszewski MC	2007	Brazil	14/12	18/15	8.54±2.92	8.69±1.83	NA	NA	Serum	RIA	Cross-sectional study
Stylianou C	2006	Greece	7/7	10/10	19.06±5.08	19.05±5.69	18.26±2.24	22.21±2.77	Serum	RIA	Case-control study
Schmitt-Grohe S(Mild)	2006	Germany	8/14	8/14	1-43	1-40	18.94±3.8	19.74±2.85	Serum	ELISA	Case-control study
Schmitt-GroheS(Moderate)	2006	Germany	8/14	8/14	1-43	1-40	17.14±2.6	19.74±2.85	Serum	ELISA	Case-control study
Ahme ML	2004	UK	66/77	20/20	8.76±11.76	9.46±1.15	NA	NA	Serum	RIA	Case-control study
Arumugam R	1998	USA	17/10	6/6	24.76±5.22	25.45±3.57	20.71±2.72	21.5±2.19	Serum	RIA	Case-control study

F, female; M, male; NA, not available; RIA, radioimmunoassay; ELISA, enzyme linked immunosorbent assay; BMI, body mass index; NGT, normal glucose tolerance; AGT, abnormal glucose tolerance; CFRD, cystic fibrosis-related diabetes; IGT, impaired glucose tolerance.

### 2.6 Data collection methods used in the study

The data were gathered by two independent reviewers. The following data were obtained from each of the included studies using a designed table (1): basic study information (study design, population, year of publication, first author, etc.) (2); baseline characteristics of patients (serum/plasma leptin concentrations, age, body mass index (BMI), number, and sex); and (3) Pearson’s correlation coefficient (COR) or Spearman’s rank COR for Forced Expiratory Volume in the first second (FEV1), fat mass, BMI, and leptin concentrations. The authors that were qualified for meta-analysis were contacted by email at least twice if the study lacked crucial information. The missing information was then obtained from the authors.

### 2.7 Assessing the research quality

The Newcastle-Ottawa Scale, which is frequently used to grade the case-control and cross-sectional studies, was employed by both researchers to score the quality of every study in an independent manner. The maximum score for the case-control or cohort studies was 9 points, whereas the highest score for the cross-sectional studies was 6 points (16). The method showing the higher score denotes a better quality. The two researchers discussed their differences and reached a common agreement.

### 2.8 Statistical analysis

The data were extracted, summarized, and examined using R (ver. 4.1.3) and Stata software (11.0). The continuous variables were normalized and reported as Standardized Mean Differences (SMD) with a 95% Confidence interval (95% CI). The links between FEV1/fat mass/BMI and leptin concentrations in the CF patients were investigated in this meta-analysis using Spearman’s CORs. The standard error that is dependent on the importance of rank COR indicates that Spearman’s product-moment COR does not depend on sampling distribution. Consequently, each COR was compared with a Fisher transformation, and after conducting an investigation where transformed values were used as an input, the coefficients of correlation (CORs) were reconverted. Additionally, the relationship between FEV1/fat mass/BMI and leptin concentrations was investigated using Pearson’s COR. Many studies have described a technique for converting Pearson’s to Spearman’s COR, using the below formula:


$$r=\;2\;sin\;(r_{s}\frac{\pi }{6})$$

where Pearson’s and Spearman’s CORs are represented by r and r_s_, respectively (17). Data heterogeneity was investigated with Cochran’s Q and chi-square tests. The I^2^ statistic was employed to identify heterogeneity (i.e., 25%, 50%, and 75% that indicated a low, moderate, and high heterogeneity, respectively; while I^2^ ≥ 50% and I^2^ < 50% revealed a high and low heterogeneity between different studies, respectively). The random- and fixed-effects models were adopted when the studies showed no heterogeneity.

The cause of heterogeneity was assessed using meta-regression, subgroup, and descriptive analyses. To conduct subgroup analysis, the overall population was categorized into groups based on the severity of the condition, sex, the technique used to test the specimen, study design, and age. For sensitivity analysis, one study at a time was eliminated to examine how every study affected the overall effect size. The linear regression, Begg’s, and Egger’s tests were carried out to determine the publication bias.

### 2.9 Bioinformatics analysis

Dataset (GSE193782) was derived from the Gene Expression Omnibus database (GEO) (https://www.ncbi.nlm.nih.gov/geo/). Single-cell sequencing data from four healthy controls and three CF patients were included in the GSE193782 dataset (18). Bronchoalveolar lavage fluid (BALF) was used as the sample in the study. Basic patient data in Single-cell dataset are provided in **Supplementary Table 1**.

The “Seurat” R package (19) was employed to carry out the computational analyses of the GSE193782 dataset. Both datasets were subjected to quality control assessments using the “Seurat” R package (200< no. of feature RNA< 5,000; percent ribosomal genes > 3; mitochondrial gene percent< 20%, erythrocyte gene percentage< 0.1). The Seurat RunPCA tool was used to conduct Principal component analysis (PCA). The Seurat NormalizeData functions were employed to normalize the scRNA-seq data, and RPCA-based Seurat IntegrateData and FindIntegrationAnchors algorithms were used to merge data across different samples. Dimension reduction was accomplished using UMAP (Universal manifold approximation and projection), and Louvain clusters were examined with the help of 30 primary principal components using the Seurat FindClusters and RunUMAP functions, respectively, with a resolution of 0.8. Seurat’s FindAllMarkers tool was employed to identify the markers in every cluster, and the CellMarker (20), PanglaoDB (21), and original research articles included in both datasets were used to identify the cell types based on the markers in each cluster. Seurat’s VlnPlot, FeaturePlot, and DotPlot functions were used to visualize the distribution of genes and their expression. The “select” function of the R software was used to assess the LEP and LEPR mRNA expression levels in the GSE193782 samples.

## 3 Results observed in the study

### 3.1 Study selection

The flowchart for the screening procedure has been presented in **Figure 1**. In this study, a total of 447 references were reviewed and included in this meta-analysis after removing duplicate references. Moreover, 217 of 447 articles were excluded during the ti/ad screening because they were beyond the parameters included in this study. After screening the full-texts (n = 26; n = 2 were not included since the full-text was unavailable, lacked a control set (n =5), did not evaluate leptin concentrations as a biomarker, which was within the scope of this review (n = 4), or the study did not include human samples (n = 1). Thus, 14 papers (9, 11–13, 22–31) were identified, which included the serum/plasma leptin concentrations, with 14 studies reporting the plasma leptin concentrations, whereas 15 studies reported the serum leptin concentrations. **Table 1** lists the 9 cross-sectional studies and 20 case-control studies that were included here. The data linked to age, sex, leptin concentrations, FEV1, severity, and BMI were summarized in **Table 2**. 12 studies reported Pearson’s or Spearman’s CORs between leptin concentrations and BMI, FEV1, and fat mass.

**Table 2:** Participants’ characteristics of included studies.

Author	Year	leptin						R	NOS
		Case		Control					
Arumugam R(male)	1998	2.2±1.4	ng/mL	3.4±1.2		ng/mL			5
Arumugam R(female)	1998	7.1±4.1	ng/mL	5.4±3.7		ng/mL			5
Ahme ML (male)	2004	1.07±0.49	ng/mL	1.02±0.5		ng/mL		R1= 0.45	5
Ahme ML (femle)	2004	2.11±0.86	ng/mL	1.34±0.44		ng/mL		R1= 0.28	5
Schmitt-Grohe S(mild)	2006	5.22±6.82	pg/mL	6.78±9.9		pg/mL		R2= 0.351	8
Schmitt-GroheS(moderate)	2006	6.96±11	pg/mL	6.78±9.9		pg/mL		R2= 0.351	8
Stylianou C(male)	2006	11.27±1.54	ng/mL	8.78±5.59		ng/mL			7
Stylianou C(female)	2006	21.57±7.68	ng/mL	14.89±5.71		ng/mL			7
Boguszewski MC (male)	2007	2.07±0.79	ng/mL	3.07±1.28		ng/mL		R1= 0.6	4
Boguszewski MC (female)	2007	2.71±0.86	ng/mL	5±2.95		ng/mL		R1= 0.6	4
Cohen RI (mild)	2008	8.4±1.8	pg/mL	8.2±2		pg/mL		R1=0.56; R2=0.4; R3=0.34	7
Cohen RI (moderate)	2008	8.1±1.7	pg/mL	8.2±2		pg/mL			7
Cohen RI (severe)	2008	2.4±2	pg/mL	8.2±2		pg/mL			7
Speeckaert MM (male)	2008	2.46±2.24	ug/L						6
Speeckaert MM (female)	2008	4.4±3.3	ug/L						6
Olveira G(male)	2012	3.9±4.4	ng/mL	3.2±1.5		ng/mL		R1=0.72; R2=0.47; R3=-0.14	5
Olveira G(female)	2012	12.9±11	ng/mL	11.2±4.1		ng/mL			5
Ziai S(NGT)(male)	2012	2.14±1.9	pg/mL	1.57±0.73		pg/mL		R1=0.82; R2=0.32	7
Ziai S(AGT)(male)	2012	1.81±1.56	pg/mL	1.57±0.73		pg/mL			7
Ziai S(CFRD)(male)	2012	4.1±3.66	pg/mL	1.57±0.73		pg/mL			7
Ziai S(NGT)(female)	2012	9.24±5.2	pg/mL	9.1±6.33		pg/mL			7
Ziai S(AGT)(female)	2012	9.17±4.13	pg/mL	9.1±6.33		pg/mL			7
Ziai S(CFRD)(female)	2012	6.87±4.57	pg/mL	9.1±6.33		pg/mL			7
Monajemzadeh M(male)	2013	27.16±33.8	ug/L	5.15±6.41		ug/L			7
Monajemzadeh M(female)	2013	13.85±16.8	ug/L	8.37±9.08		ug/L			7
Nowak JK	2020							R3=-0.1	
Polito R	2019	9.2±11.2	ng/mL	12±6.1		ng/mL			8
Granados A(NGT)	2021	14.62±14.3	ug/L	27.7±21.01		ug/L		R1=0.31; R2=0.3	7
Granados A(AGT)	2021	12.05±16.3	ug/L	27.7±21.01		ug/L			7
Granados A(CFRD)	2021	7.9±7.3	ug/L	27.7±21.01		ug/L			7
Galiniak S(male)	2022	12.17±5.65	ng/mL	4.97±1.58		ng/mL		R1=0.5025; R2=0.649; R3=-0.494	8
Galiniak S(female)	2022	15.78±7.85	ng/mL	7.43±2.7		ng/mL			8

NOS, Newcastle-Ottawa Scale; R1, correlation coefficient between leptin concentrations and fat mass; R2, correlation coefficient between leptin concentrations and BMI; R3, correlation coefficient between leptin concentrations and FEV1; NGT, normal glucose tolerance; AGT, abnormal glucose tolerance; CFRD, cystic fibrosis-related diabetes; IGT, impaired glucose tolerance.

### 3.2 Assessing literature quality

The Newcastle-Ottawa Scale was based on 3 categories, i.e., comparability (0-2 points), selection (0-4 points), and outcome (0-3 points); scores of 0-3,4-6 and 7-9 were considered to indicate low- (grade C), medium- (grade B) and high-quality (grade A) studies, respectively. Every study exhibited relatively high quality (grade B and grade A), as shown in **Table 2**. Thus, we did not conduct further analysis according to literature quality.

### 3.3 Meta-analysis

#### 3.3.1 Leptin levels in CF patients

**Figure 2:**
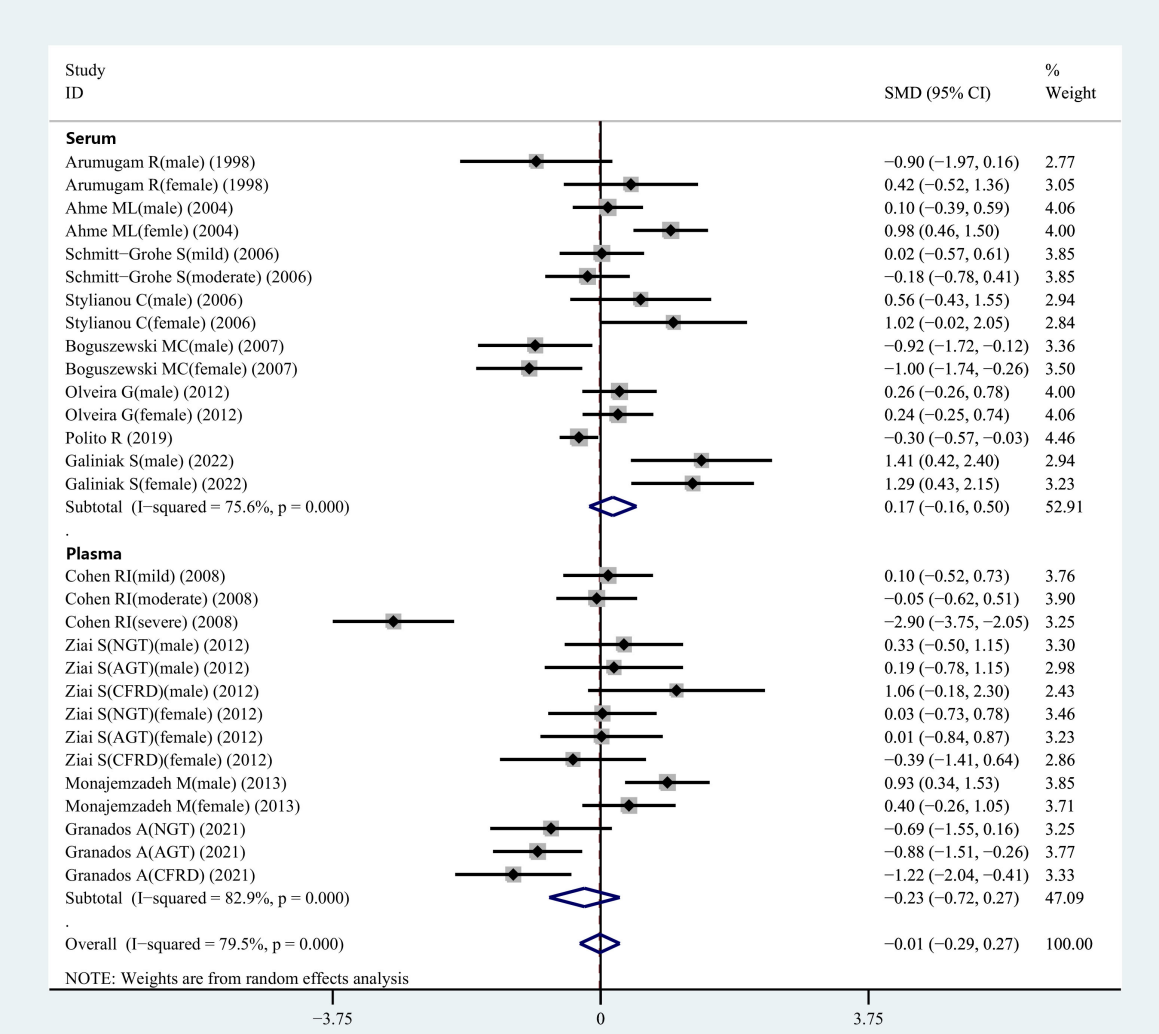
SMD forest plot and its 95%CI for serum/plasma leptin concentrations in the CF group and the control group.

Out of the 29 studies included in the review. The heterogeneity index of I^2^ was 79.5%. Hence, a random-effects model was selected for validating the reliability of the data. The meta-analysis showed that leptin concentrations between the CF patients and healthy controls did not differ significantly (SMD=-0.01, 95% CI=-0.29-0.27, P=0.935; **Figure 2**). To assess the validity of combined data, many sensitivity analyses were carried out. After independently excluding each of the included studies, no significant variations were observed in the meta-findings analysis, demonstrating its validity (**Figure 1**). The differences in the plasma/serum leptin levels in CF patients were investigated, because sample types might be a source of heterogeneity for the outcome of meta-analysis.

#### 3.3.2 Comparing the serum leptin concentrations in the CF patients and healthy controls

During the meta-analysis, 15 observational studies were analyzed to determine the relationship between serum leptin levels in CF patients and controls. Serum leptin levels did not show significant variations between CF and healthy control individuals (SMD=0.17, 95% CI=-0.16-0.50, P=0.309; **Figure 2**). Because of the heterogeneity (I^2 =^ 75.6%), a random-effects model was selected for further analyses.

#### 3.3.3 Comparing the plasma leptin concentrations between the CF patients and healthy individuals

**Figure 2** displays the results of a pooled analysis of plasma leptin levels in CF patients. The results demonstrated no significant variation in plasma leptin levels between CF patients and healthy controls (SMD=-0.23, 95% CI=-0.72-0.27, P=0.369; **Figure 2**). Owing to the presence of heterogeneity (I^2 =^ 82.9%), a random-effects model was employed for further analyses.

### 3.4 Subgroup analysis of serum leptin concentrations

#### 3.4.1 Age

Four studies were carried out for comparing the serum leptin concentrations in children. The results indicated no significant variation in the serum leptin levels between the healthy and CF children (SMD=-0.17, 95% CI=-1.07-0.73, P=0.711, **Table 3**). Similar findings were reported when blood leptin levels in healthy and CF patients(adults) were evaluated in seven studies (SMD=0.13, 95% CI=-0.25-0.52, P=0.494, **Table 3**).

**Table 3:** Subgroup analyses of leptin concentrations in CF and controls.

Subgroup analysis of plasma levels(n)	SMD(95% CI)	P-value	I^2^(%)	*P*_h_	Subgroup analysis of serum levels(n)	SMD(95% CI)	*P*-value	I^2^(%)	*P*_h_
Overall (14)					Overall (15)				
Age									
Adult (9)	-0.20(-0.86,0.46)	0.5533	82.90%	<0.0001	Adult (7)	0.13(-0.25,0.52)	0.494	60.30%	0.019
Children (2)	0.68(0.16,1.21)	0.011	30.00%	0.232	Children (4)	-0.17(-1.07,0.73)	0.711	88.20%	<0.0001
Mixture (3)	-0.93 (-1.36,-0.50)	<0.0001	0.0%	0.665	Mixture (4)	0.56(-0.21,1.33)	0.309	77.5%	0.004
Sex									
Female (4)	0.10(-0.30,0.49)	0.630	0.0%	<0.0001	Female (6)	0.47(-0.17,1.12)	<0.0001	79. 0%	<0.0001
Male (4)	0.67(0.26,1.08)	0.001	0.0%	0.428	Male (6)	0.08 (-0.49,-0.66)	<0.0001	71.50%	0.004
Mixture (6)	-0.91(-1.71,-0.11) ,	0.026	87.2%	<0.0001	Mixture (3)	-0.24(-0.47,-0.01)	<0.0001	0.0%	0.614
Assay approaches									
ELISA (5)	-0.27(-1.35,0.80)	0.619	52.10%	<0.0001	ELISA (7)	0.27(-0.14,0.68)	0.896	74.50%	0.001
RIA (9)	-0.24(-0.68,0.19)	0.268	55.90%	0.02	RIA (8)	0.04 (-0.55,0.63)	0.196	78.90%	<0.0001
Design									
Cross-sectional study (3)	-0.93(-1.36,-0.50)	0.922	0.0%	0.665	Cross-sectional study (6)	0.19(-0.50,0.87)	0.597	82.90%	<0.0001
Case-control study (11)	-0.03(-0.60,0.54)	<0.0001	83.30%	<0.0001	Case-control study (9)	0.17(-0.21,0.55)	0.389	70.80%	0.001

Ph: P_heterogeneity;_ RIA, radioimmunoassay; ELISA, enzyme linked immunosorbent assay.

#### 3.4.2 Gender

Subgroup analysis was also carried out regarding gender as it was recorded in several studies. Six studies reported serum leptin levels in male CF patients, and it was observed that the male CF patients displayed similar values to the healthy male controls (SMD=0.08, 95%CI=-0.49-0.66, P=0.775, **Table 3**). Six studies observed no significant variations in serum leptin levels between healthy and CF female patients (SMD=0.47, 95% CI=-0.17-1.12, P=0.149, **Table 3**).

#### 3.4.3 Assay techniques

Subgroup analysis was carried out using different detection techniques for different samples since variations in the serum leptin level detection methods could lead to errors in the outcome analysis. An Enzyme-linked immunosorbent assay (ELISA) was utilized in seven studies to assess serum leptin levels. The results showed no significant variations in serum leptin concentrations between the CF and healthy control individuals (SMD=0.27, 95% CI=-0.14-0.68, P=0.196, **Table 3**). A radioimmunoassay was utilized for calculating the serum leptin levels in 8 studies, and the findings indicated no significant changes in the serum leptin concentrations between the CF and healthy control patients (SMD=0.04, 95% CI=-0.55-0.63, P=0.896, **Table 3**).

#### 3.4.4 Research design

A matched subgroup analysis was conducted in this study since the different study designs used in various studies could affect the heterogeneity values of the results. A subgroup analysis of the cross-sectional studies showed no significant variations in the serum leptin levels between the CF and normal control individuals (SMD=0.19, 95%CI=-0.50-0.87, P=0.597, **Table 3**). A subgroup analysis of case-control studies also revealed no significant differences in the serum leptin levels between the CF patients and healthy individuals (SMD=0.17, 95% CI=-0.21-0.55, P=0.389, **Table 3**).

### 3.5 Subgroup analysis of plasma leptin concentrations

#### 3.5.1 Age

Two studies compared plasma leptin levels in CF and healthy children. The combined outcomes showed that plasma leptin levels in CF children were higher compared to healthy children (SMD=0.68, 95% CI = 0.16-1.21, P=0.011, **Table 3**). Nine studies investigated the blood leptin concentrations in CF patients(adults), and the combined results indicated similar plasma leptin concentrations between CF and healthy control individuals(adults) (SMD=-0.20, 95% CI = -0.86-0.46, P=0.553, **Table 3**).

#### 3.5.2 Gender

Four studies determined the plasma leptin concentrations in the male CF patients, and the results indicated that the CF patients(male) exhibited elevated plasma leptin concentrations compared to the healthy male controls (SMD=0.67, 95% CI = 0.26-1.08, P=0.001, **Table 3**). The results of 4 studies showed that plasma leptin concentrations in the female CF patients were similar to those displayed by the female control individuals (SMD=0.10, 95% CI = -0.30-0.49, P=0.630, **Table 3**).

#### 3.5.3 Assay approaches

An ELISA technique was used in five studies to measure plasma leptin levels. Similar results were observed between the CF patients and healthy control individuals (SMD=-0.27, 95% CI=-1.35-0.80, P=0.619, **Table 3**). The RIA technique was also used to estimate the plasma leptin levels in nine studies, and the findings indicated similar plasma leptin concentrations in CF patients and healthy control groups (SMD=-0.24, 95% CI= -0.68-0.19, P=0.268, **Table 3**).

#### 3.5.4 Research design

Cross-sectional studies were carried out where the CF patients showed significantly lower blood leptin concentrations compared to healthy control individuals (SMD=-0.93, 95% CI=-1.36 -0.50, P<0.001, **Table 3**). A subgroup analysis of case-control studies revealed no significant variations in the plasma leptin concentrations between CF patients and control individuals (SMD=-0.03, 95% CI = -0.60-0.54, P=0.922, **Table 3**).

#### 3.5.5 Glucose tolerance

**Figure 3:**
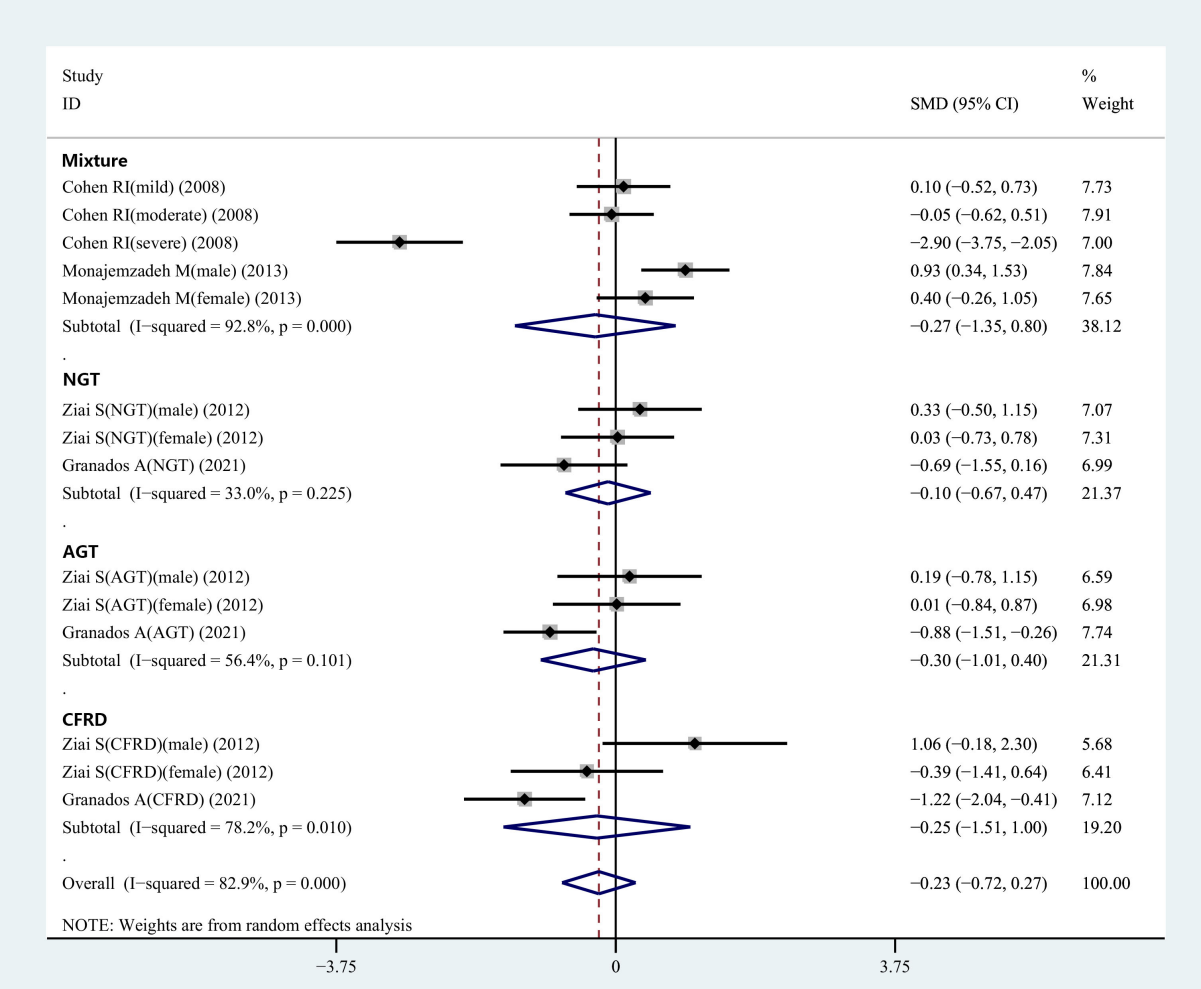
Subgroup analysis depended on the glucose tolerance status of CF patients compared to the controls displayed in the meta-analysis. NGT, normal glucose tolerance; AGT, abnormal glucose tolerance; CFRD, cystic fibrosis-related diabetes.

The information regarding glucose tolerance in CF patients was presented in two articles involving 9 studies. Subgroup analysis was carried out based on the different glucose tolerance in the CF patients. The results revealed no differences in the plasma leptin concentrations between the control and normal glucose tolerance groups, control and abnormal glucose tolerance groups, as well as control and CF-related diabetes groups (**Figure 3**).

### 3.6 Comparison of leptin levels between female and male CF patients

The data regarding the leptin levels in male and female CF patients were presented in 11 studies. Pooled analysis was conducted using the leptin levels for the two groups, and the results revealed that male CF patients exhibited considerably lower leptin levels than the female CF patients (SMD=-1.07, 95% CI=-1.52- -0.61, P<0.001, **Table 3**). Moreover, the leptin levels in healthy male and female control groups were extracted and subjected to pooled analysis. The findings revealed that normal male participants had lower leptin levels compared to normal female individuals (SMD=-1.20, 95% CI = -1.74-0.66, P<0.001, **Table 3**).

### 3.7 Meta-analysis of the relationship between the serum/plasma leptin concentrations and BMI, FEV1, and fat mass

**Figure 4:**
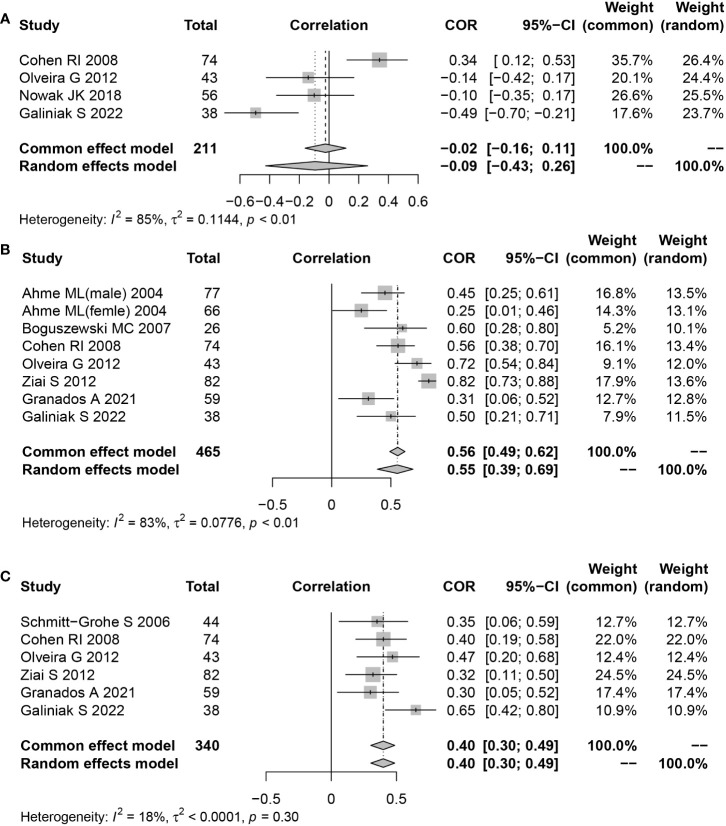
Funnel plot of effect sizes measured as correlations between serum/plasma leptin conclusions and FEV1, fat mass and body mass index. **(A)** FEV1; **(B)** fat mass; **(C)** body mass index.

The correlation between serum/plasma leptin levels and FEV1 was revealed in four studies using Pearson’s or Spearman’s CORs. Furthermore, eight studies also determined the correlation between serum/plasma leptin levels and fat mass using either Spearman’s or Pearson’s CORs. Serum/plasma leptin concentrations and BMI were correlated with each other in six studies using either Pearson’s or Spearman’s CORs. A “meta” R software was used for conducting the meta-analysis using the serum/plasma leptin concentrations, in addition to the BMI, FEV1, and fat mass in CF patients. The analysis showed that the effect size for the serum/plasma leptin concentrations and FEV1 was -0.09 (95% CI = -0.43-0.26, P=0.607) (**Figure 4**). The effect size for the fat mass and serum/plasma leptin concentration was 0.55 (95% CI=0.39-0.69, P<0.001) (**Figure 4**). Furthermore, the effect size for BMI and serum/plasma leptin level was 0.40 (95% CI=0.30-0.49, P<0.001) (**Figure 4**).

### 3.8 Meta-regression

All included studies had an I^2^ value of 79.5%, indicating a high level of heterogeneity. Therefore, we explored the possible sources of heterogeneity using meta-regression. **Table 4** showed the meta-regression of serum/plasma leptin concentrations. The meta-regression of serum leptin concentration indicated P values of 0.679, 0.198, 0.500, 0.990 for covariates of age, sex, assay approaches, research design, respectively. The meta-regression of plasma leptin concentration indicated P values of 0.599, 0.089, 0.927, 0.164 for covariates of age, sex, assay approaches, research design, respectively. This information suggested that the former factors had no significant effect on heterogeneity.

**Table 4:** Meta-regression analysis of variables predicting serum and plasma levels of leptin.

Variables	R	Adjusted R^2^	*P*
Age			
Serum	-0.065	-0.103	0.679
Plasma	0.114	-0.078	0.599
Sex			
Serum	0.324	0.107	0.198
Plasma	0.089	0.195	0.089
Assay approaches			
Serum	-0.272	-0.099	0.500
Plasma	0.053	-0.109	0.927
Design			
Serum	-0.0003	-0.123	0.990
Plasma	-0.452	0.113	0.164

### 3.9 Publication bias

**Figure 5:**
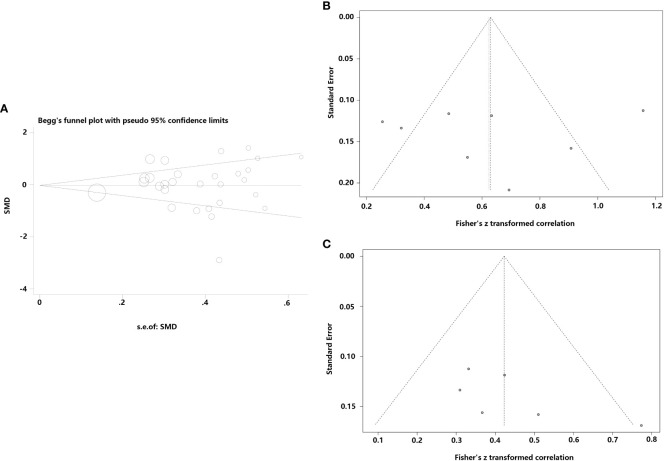
Funnel plots were employed to assess the publication bias among the included studies examining the relationship of leptin concentrations with CF. **(A)** Funnel plot of the serum/plasma leptin concentrations in patients with CF versus the control group. **(B)** Funnel plot of the correlation coefficient between serum/plasma leptin concentrations and fat mass. **(C)** Funnel plot of the correlation coefficient between serum/plasma leptin concentrations and body mass index.

Studies examining the correlation between CF and leptin concentrations, which seemed symmetrical, were investigated using funnel plots to determine the likelihood of publication bias. Several CF-related studies revealed that neither Begg’s (P=0.722) nor Egger’s (t=0.27, P=0.789) tests suffered a publication bias (**Figure 5**). Additionally, a funnel plot was generated to assess the publication bias of 2 meta-analyses using combined CORs (leptin concentrations *vs* fat mass; and leptin concentrations *vs* BMI). The funnel plots showed no publication bias and appeared systematic (P=0.145, P=0.869; **Figure 5B**). Only four studies showed a correlation between leptin concentrations and FEV1; hence, a publication bias analysis was not conducted.

### 3.10 Bioinformatics analysis

**Figure 6:**
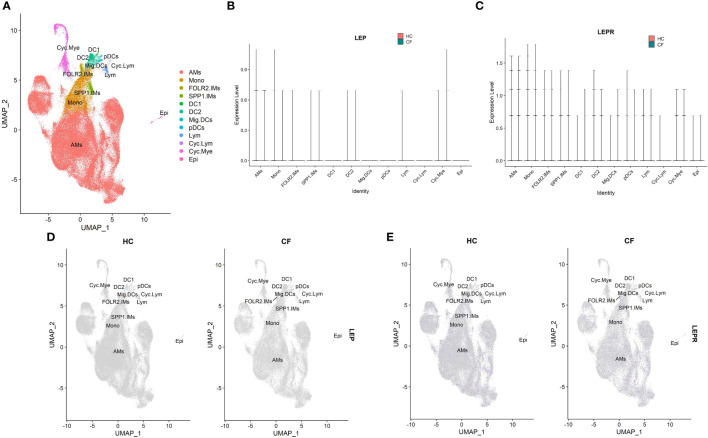
The scRNA-seq analysis in the GSE193782(CF = 3, control = 4). **(A)** Cell type annotation. **(B)** The expression of LEP mRNA in different cell groups. **(C)** The expression of LEPR mRNA in different cell groups. **(D)** Feature plot of LEP mRNA in CF patients and controls. **(E)** Feature plot of LEPR mRNA in CF patients and controls. The 12 major cell types: AMs, alveolar macrophages; Cyc. Mye, myeloid cycling cells; Cyc. Lym, lymphoid cycling cells; FOLR2.IMs , FOLR2 interstitial macrophages; SPP1.IMs, SPP1 interstitial macrophages; Mono, monocytes; DC1, dendritic cells 1; DC2, dendritic cells 2; Mig. DC, migratory dendritic cells; pDCs, plasmacytoid dendritic cells; Lym, lymphocytes; Epi, epithelial cells.

**Supplementary Table 1** summarizes the information derived from the single-cell RNA-seq dataset (GSE193782) involving CF patients. Leptin acts through a particular homodimeric receptor known as the leptin receptor (LEPR) (32). Hence, the mRNA expression levels of LEP and LEPR were analyzed. The results showed that the different cell types displayed low leptin (LEP) and leptin receptor (LEPR) mRNA expression levels in alveolar lavage fluid, without specific distribution (**Figures 6A–E**). However, no statistical difference was observed in the leptin and LEPR mRNA expression levels between the CF patients and healthy individuals (both P>0.05).

## 4 Discussion

This is the first study to report the results of meta-analysis and meta-regression of the association between leptin concentrations and cystic fibrosis. 14 articles were found to be acceptable for the meta-analysis, which included 919 CF patients and 397 healthy individuals. Two major phenomena were identified in this study. Firstly, in comparison to healthy non-CF individuals, no significant increase or decrease was observed in the serum leptin levels collected from CF patients. No significant variations were observed in serum leptin levels between the healthy participants and CF patients after subgroup analysis by age, research design, sex, and detection technique. Secondly, no significant variations were observed in plasma leptin levels between the CF patients and healthy individuals. The results of subgroup analysis based on age, sex, research design type, detection technique, and glucose tolerance status, indicated that the plasma leptin levels of CF patients that were determined using various study design types, detection methods, and glucose tolerance status, were not statistically different from those exhibited by the control individuals. Furthermore, plasma leptin levels in male CF patients and children with CF were elevated in comparison to those displayed by control participants. However, after analyzing the primary data, the children’s plasma leptin expression data were derived from a single institution (24). A large sample size needs to be used to confirm the aforementioned findings. Secondly, leptin levels were seen to be significantly decreased in male CF patients compared to female CF patients as well as in normal male individuals compared to normal female subjects. Finally, leptin levels appeared to be favorably connected with fat mass and BMI but not with the FEV1 of CF patients. The results of the single-cell sequencing analysis indicated no significant variation in leptin levels between healthy individuals and CF patients. Leptin levels appeared to have a higher correlation with sex, fat mass, and BMI than with CF.

The obese gene (*ob* gene), located on the long arm of human chromosome 7 (7q31), encodes the protein leptin (33). Initially, researchers thought that leptin was synthesized and secreted by the body’s white adipose tissues, infiltrated the bloodstream, and affected the hypothalamus to control energy metabolism *via* the neuropeptide Y transmitter system and α-melanocyte stimulating hormone (α-MSH) system (34). Leptin is a component of the fat-islet endocrine axis and controls endocrine insulin production (35). Earlier studies have indicated that leptin levels reflect a lower body fat level in anorexic individuals, or higher body fat levels in obese individuals (36, 37). Several studies have demonstrated that leptin protein is found in the body’s fats, along with in different organs and tissues in the body. Leptin is synthesized in the alveolar type II cells and bronchial epithelial cells, indicating that leptin may control the immune responses and inflammation as well as influence the onset and progression of lung disorders (38, 39). Inconsistent data regarding the leptin levels in CF patients were found in the included literature, which suggested that leptin production was dysregulated in CF patients. Studies conducted in a mouse CF-model mouse demonstrated that increased caloric intake and loss of leptin signaling and metabolic control mechanisms could cause an increase in fat storage ability and body length in mice (40). Additionally, aberrant immunological responses and susceptibility to infections, which are commonly found in CF, are also influenced by leptin imbalance (41). It should be mentioned that leptin plays a crucial role in the growth and regulation of the redox system. Elevated levels of leptin may cause the release of reactive oxygen species and enhance inflammation, which may be one of the factors contributing to excessive oxidative stress in CF (12).

Nevertheless, the pooled findings observed in this review indicated that there were no significant variations in serum/plasma leptin levels between CF patients and normal, healthy individuals. No discernible differences were observed in leptin levels between the groups even after subgroup analysis based on sex, age, detection method, type of experimental design, etc. The results could be attributed to variations in leptin levels among various CF groups. Galiniak et al. (12) observed that compared to healthy controls, leptin levels were lower in children and young CF patients while opposite results were obtained by Monajemzadeh et al. (24) who observed lower leptin levels in healthy individuals. According to Nowak et al.’ study (23), children under the age of 16 showed higher leptin levels than those displayed by elderly patients. Although an age-based subgroup analysis was conducted in this study, the results indicated that there were no significant differences in leptin levels between CF children/adults and control children/adults. We only may speculate the kind of CFTR gene mutation could have an impact on the disease’s severity and might be linked to leptin levels. Mekus et al. (42) demonstrated that loci in the partially imprinted 3’ region of CFTR, like the Silver-Russel-Syndrome candidate gene area and LEP, that control the food intake, stature, and energy homeostasis, also affect disease manifestation in CF. Very few studies offered information regarding the kind of CFTR gene mutation, hence additional subgroup analysis based on the type of gene mutation could not be conducted in this study. Leptin levels may also be impacted by the severity of the disease, although only two of the included studies (28, 31) classified CF patients into mild, moderate, and severe categories. In their study, Schmitt-Grohe et al. (31) revealed that only patients with mild CF exhibited lower serum leptin levels than healthy individuals, whereas Cohen et al. (28) observed that severe CF patients showed lower serum leptin levels in comparison to healthy individuals Both researchers concluded that the severity of the CF patients could be linked to lowered leptin levels. The relationship between illness severity and leptin levels, however, could not be determined due to the small sample sizes used in both articles. Notably, the results in this study revealed that normal female individuals had higher leptin levels than normal male participants, while the female CF patients showed significantly higher leptin levels compared to the male CF patients. The outcomes revealed that leptin levels varied by gender. Women’s distinctive physiological features, such as their breasts, buttocks, and lower limbs, have larger fat contents than those of men. Additionally, their leptin synthesis rate per unit of adipose tissue was higher compared to that displayed by men (43). The release and secretion of leptin are reportedly inhibited by testosterone, and the inhibitory effect is dependent on leptin protein and mRNA levels (44). The results derived from *in-vitro* adipose tissue cultures revealed that estradiol enhanced the secretion and release of leptin in females, but not in males (45). The findings of the meta-analysis in this review were in agreement with those presented in the earlier studies.

This study showed that leptin levels in CF patients were comparable to those in controls, unrelated to FEV1, and positively linked to mass and BMI. According to the single-cell sequencing data, leptin-related mRNA was expressed at extremely low levels in the cells of the alveolar lavage fluid and was also not widely distributed in immune cells. These results indicated that the physiological regulation of leptin is related to body fat in chronic diseases. The results were similar to the findings presented by Arumugam et al. (13), who observed that the serum leptin is mainly synthesized in the white adipose tissues and is related to the body fat content.

CF-related diabetes (CFRD) was regarded as the most frequent extrapulmonary consequence of cystic fibrosis (46). CFRD is primarily caused by insulin deficiency, however, the fluctuations in the insulin resistance level associated with the acute and chronic disease could also lead to this disease (47). *In vitro* animal studies indicated that insulin positively affected leptin secretion, while a few other studies indicated that it affected the mRNA expression of the LEP gene (25, 48). Ziai et al. (25) observed that plasma leptin levels in CF patients were significantly linked with glucose-stimulated insulin secretion, even after they had adjusted the confounding variables like gender and fat mass. This suggested that insulin could play a role in controlling leptin levels in CF patients. Subclinical inflammation, however, is another sign of CF in addition to inflammation caused by repeated bacterial infections (49). Many researchers carried out cellular and animal studies and observed that inflammation-related cytokines such as Tumor necrosis factor-α and Interleukin (IL)‐6 increased leptin levels (28). As a result, two conditions were associated with decreased insulin secretion in CF patients: low leptin levels due to impaired insulin secretion; or high leptin levels in the subclinical inflammatory state. The interaction between these two states could account for the similarity in leptin levels between healthy controls and CF patients with reduced glucose tolerance. Leptin levels in CF patients and healthy individuals were identical. This raises the question- Is this phenomenon associated with impaired insulin secretion? In this study, it was hypothesized that this could be related, however, some studies stated that CF patients could be asymptomatic for several years, hence, it was recommended that CF screening should be conducted at 10 years of age. Even though an oral glucose tolerance test is advised, it is generally known that it cannot detect the early anomalies in how the body handles glucose and that this will indicate that opportunities for early detection would be lost (50).

In a meta-analysis, heterogeneity between studies is typically correlated with demographic characteristics, disease severity, experimental design, the caliber of the included research, and other parameters. The results in this study showed no significant variations across various studies that examined the correlation between CF and leptin levels. Subgroup and meta-analysis were carried out to investigate the underlying cause of heterogeneity. No heterogeneity was observed in the meta-regression analysis related to age, sex, assay techniques, or research design. However, no obvious causes of heterogeneity were discovered. These elements may also enhance heterogeneity and impair the accuracy of this meta-analysis. Additionally, the “leave-one-out” sensitivity analysis found that no individual study was responsible for the high heterogeneity. Thus, it was hypothesized in this study that several factors could lead to heterogeneity, such as variations in blood collection techniques, storage, measurement, and experimental procedures, as well as types of gene mutations, dietary and lifestyle preferences, illness severity, and other underlying confounding variables.

This is, to our knowledge, the first meta-analysis study that has examined leptin levels in CF individuals. However, this meta-analysis study has a few limitations. Firstly, it is difficult to establish a causal association since all publications included in this meta-analysis were observational studies and lacked multi-center and large-cohort RCT trials. Numerous gaps were observed in studies that investigated the effect of age, illness severity, and genotype on leptin concentrations. The relationship between leptin levels and CF needs to be verified by longitudinal studies. Secondly, the high-throughput sequencing data related to blood samples or lung tissues were not available for validation in the public databases, and the meta-analysis used only single-cell sequencing datasets for validating the data validated using bioinformatics. Thirdly, innovative therapies that alter the negative consequences of CFTR mutations at the molecular level, such as CFTR modulators, are gaining a lot of acceptance as standard therapies. Further research is needed to determine whether leptin concentrations observed in this meta-analysis study would persist even after CFTR modulator therapy.

## 5 Conclusions

The pooled results of different observational studies revealed that leptin concentrations were not significantly different between CF patients and healthy individuals. Furthermore, leptin levels were potentially related to gender, fat mass, and BMI. The association between leptin levels and clinical outcomes in CF patients is still questionable based on the available data; therefore, an in-depth study is required in the future to establish the relationship and explain the underlying mechanisms.

## Data availability statement

The original contributions presented in the study are included in the article/**Supplementary Material**. Further inquiries can be directed to the corresponding author.

## Author contributions

Data curation: JH and HQ. Formal analysis: HL and PZ. Methodology: HL and HQ. Project administration: HL. Resources: JH and PZ. Supervision: HQ. Validation: JH. Writing-original draft: JH. All authors contributed to the article and approved the submitted version.

## Supplementary material

Supplementary Figure 1 Sensitivity analysis of studies on leptin concentrations for CF patients versus controls.
