# Supplementary figures and images for: Lack of association between leptin concentrations and cystic fibrosis: A meta-analysis and regression
Source: Front Endocrinol (Lausanne). 2023 Mar 13;14:1126129. doi: 10.3389/fendo.2023.1126129 (PMC10040884; doi:10.3389/fendo.2023.1126129)

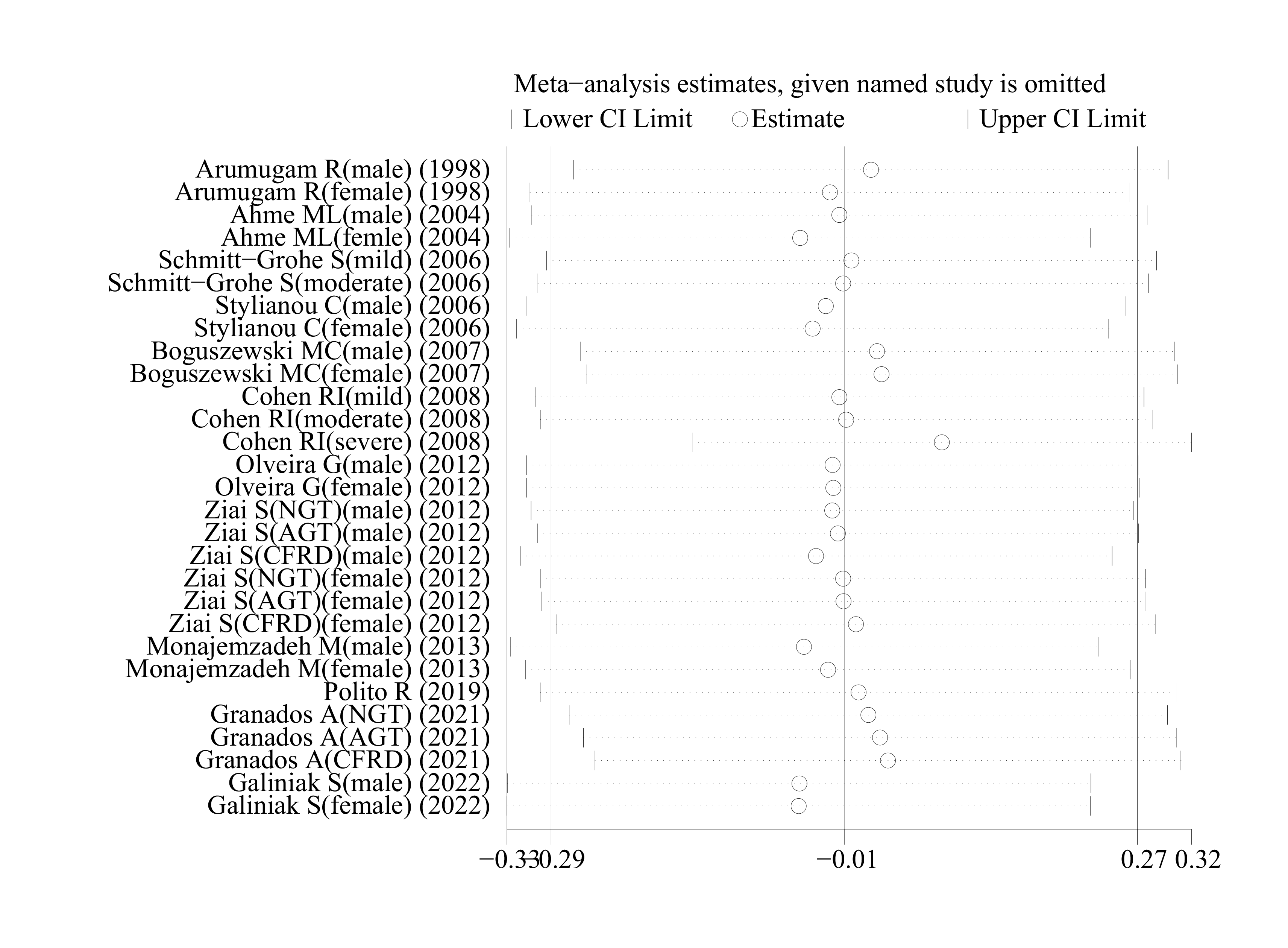

Supplement: Supplementary Figure 1 — Sensitivity analysis of studies on leptin concentrations for CF patients versus controls. [file Image_1.tif]
