# Supplementary material for: Lack of association between leptin concentrations and cystic fibrosis: A meta-analysis and regression
Source: Front Endocrinol (Lausanne). 2023 Mar 13;14:1126129. doi: 10.3389/fendo.2023.1126129 (PMC10040884; doi:10.3389/fendo.2023.1126129)
Supplement: Supplementary file 2 [file Table_1.docx]

**Supplementary Table1.Demographic characteristics for recruited subjects.**

| Characteristic | CF (n = 3) | HC (n = 4) |
| --- | --- | --- |
| Sex, female %(n) | 33% (1) | 50% (2) |
| Average age, years (SD) | 28 + 4.6 | 29 ± 6.1 |
| FEV1, per cent predicted (SD) | 89.8±11 |  |
| Pa colonization %(n) | 67% (2) |  |
| Staph aureus colonization %(n) | 33% (1) |  |
| F508del/F508del%(n) | 100% (3) |  |
| CFTR modulator use (within 12 months) %(n) | 0% (0) |  |
| Dornase alpha use %(n) | 100% (3) |  |
| Hypertonic saline use %(n) | 67% (2) |  |
| Azithromycin use %(n) | 67% (2) |  |
| Inhaled antibiotic use %(n) | 0% (0) |  |
| Oral/lV antibiotics within 6 months %(n) | 0% (0) |  |
| Pancreatic insufficiency%(n) | 100% (3) |  |
| CFRD %(n) | 0% (0) |  |
| CF liver disease | 0% (0) |  |

FEV1: Forced Expiratory Volume in the first second; SD: standard deviation; CFTR: cystic fibrosis transmembrane conductance regulator; CF: cystic fibrosis; CFRD: cystic fibrosis-related diabetes
